# Genome-Wide DNA Changes Acquired by *Candida albicans* Caspofungin-Adapted Mutants

**DOI:** 10.3390/microorganisms11081870

**Published:** 2023-07-25

**Authors:** Jeffrey Zuber, Sudisht K. Sah, David H. Mathews, Elena Rustchenko

**Affiliations:** Department of Biochemistry and Biophysics, University of Rochester Medical Center, Rochester, NY 14642, USA; jzuber@gmail.com (J.Z.); sudisht_sah@urmc.rochester.edu (S.K.S.); david_mathews@urmc.rochester.edu (D.H.M.)

**Keywords:** *Candida albicans*, adaptation to caspofungin, DNA profiling, genome-wide mutations, prevalence of single-nucleotide substitutions

## Abstract

Drugs from the echinocandin (ECN) class are now recommended ‘front-line’ treatments of infections caused by a prevailing fungal pathogen, *C. albicans*. However, the increased use of ECNs is associated with a rising resistance to ECNs. As the acquisition of ECN resistance in *C. albicans* is viewed as a multistep evolution, determining factors that are associated with the decreased ECN susceptibility is of importance. We have recently identified two cohorts of genes that are either up- or downregulated in concert in order to control remodeling of cell wall, an organelle targeted by ECNs, in laboratory mutants with decreased ECN susceptibility. Here, we profiled the global DNA sequence of four of these adapted mutants in search of DNA changes that are associated with decreased ECN susceptibility. We find a limited number of 112 unique mutations representing two alternative mutational pathways. Approximately half of the mutations occurred as hotspots. Approximately half of mutations and hotspots were shared by ECN-adapted mutants despite the mutants arising as independent events and differing in some of their phenotypes, as well as in condition of chromosome 5. A total of 88 mutations are associated with 43 open reading frames (ORFs) and occurred inside of an ORF or within 1 kb of an ORF, predominantly as single-nucleotide substitution. Mutations occurred more often in the 5′-UTR than in the 3′-UTR by a 1.67:1 ratio. A total of 16 mutations mapped to eight genomic features that were not ORFs: Tca4-4 retrotransposon; Tca2-7 retrotransposon; lambda-4a long terminal repeat; mu-Ra long terminal repeat; MRS-7b Major Repeat Sequence; MRS-R Major Repeat Sequence; RB2-5a repeat sequence; and tL (CAA) leucine tRNA. Finally, eight mutations are not associated with any ORF or other genomic feature. Repeated occurrence of single-nucleotide substitutions in non-related drug-adapted mutants strongly indicates that these DNA changes are accompanying drug adaptation and could possibly influence ECN susceptibility, thus serving as factors facilitating evolution of ECN drug resistance due to classical mutations in *FKS1*.

## 1. Introduction

The unicellular budding fungus *Candida albicans* lives as part of normal human gut and genital microbiota, but is a prevalent opportunistic pathogen in immune-compromised individuals [1]. There is a limited choice of three classes of anticandidal drugs that are currently available for therapeutic treatment of invasive infections caused by *C. albicans* [2]. Compounds from the echinocandin (ECN) class (caspofungin, anidulafungin and micafungin) that are now recommended “front-line” treatments have low toxicity, high efficacy, and limited drug–drug interactions [2,3]. However, the increased use of ECNs is associated with low-level of persistent resistance to ECNs that is slowly increasing [1,4,5]. The generally recognized mechanism of ECN clinical resistance involves point mutations in the essential *FKS1* gene (orf19.2929), encoding a catalytic sub-unit of the 1,3-β-glucan synthase complex. Mutations are clustered in two “hotspot” regions, HS1 and HS2, encompassing residues from 641 to 649, and from 1345 to 1365, respectively [6].

Because of limited therapeutic choices to treat candidiasis, combined with increasing antifungal resistance, it is of high importance to better understand how *C. albicans* develops drug resistance, in particular to ECNs [2]. In this regard, the work of different laboratories demonstrated that *C. albicans* and related *Candida* species possess mechanisms independent of *FKS1* mutations that can decrease susceptibility to ECNs, although these mechanisms do not confer clinical resistance. Various genes acting to decrease ECN susceptibility were reported (reviewed in [7]). These genes are thought to be of importance, as acquisition of clinical resistance is now regarded as a multistep evolution in which gradually decreasing susceptibility ultimately leads to the appearance of an *FKS1* mutation conferring resistance (reviewed in [8]). However, in the above approach, there is a lack of studies of genome-wide DNA changes that could be associated with decreased ECN susceptibility in *C. albicans*.

We earlier set about to search for genetic features that enable the evolution of ECN resistance in *C. albicans*. We took a distinct approach to generate caspofungin-adapted mutants in vitro by direct selection on plates supplemented with lethal amounts of caspofungin. Care was taken that the mutants derive from parallel experiments as independent mutational events [9]. These mutants typically adapt to grow in the presence of certain amounts of caspofungin in the absence of *FKS1* mutations and having their caspofungin minimum inhibitory concentrations (MICs) increased two- to eight-fold [9]. Such adaptation is called here also tolerance. Adaptation of some mutants relies upon aneuploidy of chromosome 5 (Ch5), whereas other adapted strains remain euploid [9]. We have previously used RNA-seq to analyze the genome-wide transcriptional profiles of caspofungin-adapted mutants either lacking one copy of chromosome Ch5 or having no ploidy change. We characterized a total of 15 genes that are distributed non-randomly over two different chromosomes. These genes are simultaneously upregulated on Ch2, a total of five genes, and down-regulated on Ch5, a total of 10 genes, across all adapted mutants in order to control the cell wall of these mutants for adaptation to ECNs [7,10]. Here, we continue our search for the features that are implicated with adaptation to ECN drugs.

Occurrence of genomic changes in *C. albicans* was previously studied in time series of clinical isolates that were collected from patients with oropharyngeal candidiasis treated with azole fluconazole. Various DNA changes were found in this study including copy-number variations, single nucleotide polymorphisms, loss of heterozygosity and aneuploidies [11]. A recent review summarized studies of the dynamics of genomic changes serving to colonize various host niches or adapt to diverse selection pressures, also focusing on the effects of *C. albicans* treatment with fluconazole. The reviewed genomic changes included point mutations, loss of heterozygosity, ploidy shifts and aneuploidy [12]. However, to the best of our knowledge, DNA changes that occur in cells with reduced susceptibility to ECNs were yet to be studied.

Here, we used DNA-seq to analyze DNA profiles of two mutants of each type, either lacking one copy of chromosome Ch5 or euploid, in isogenic parent/mutant pairs in an attempt to elucidate genome-wide DNA changes that could potentially have roles in adaptation to caspofungin. We find evidence of an underlying genome-wide mutagenesis that is coupled with caspofungin adaptation. Adapted strains harbor a limited number of mutations and mutational hotspots (not related to *FKS1* hot-spots) genome-wide. In addition, the four caspofungin-adapted strains detail two alternative mutational pathways. Despite these adapted mutants deriving from independent mutational events, half of the DNA changes and hotspots are shared, with mutations arising predominantly as single-nucleotide substitutions inside of ORFs or within 1 kb of ORFs, a striking contrast to the large spectrum of various mutations that occur in response to *C. albicans* exposure to fluconazole. We interpret these data as a strong indication of a non-random mutagenesis accompanying caspofungin adaptation. While our findings need further study, we suggest that at least some of the DNA changes can be candidates for influencing ECN susceptibility and can potentially serve as factors facilitating evolution of ECN drug resistance due to classical mutations in *FKS1*.

## 2. Materials and Methods

### 2.1. Strains, Media, and Growth Conditions

*C. albicans* caspofungin-adapted mutants used in this study (Table 1) were generated by exposing either a reference strain SC5314 or a clinical isolate JRCT1 to caspofungin in parallel experiments using three different concentrations of caspofungin (60 ng/mL; 160 ng/mL, and 200 ng/mL) [9], as reflected in the mutants’ names (Table 1). Specifically, approximately 1 × 10^6^ cells per plate were plated on synthetic dextrose medium (SD) [9] supplemented with lethal concentrations of caspofungin and incubated at 37 °C. Grown colonies were randomly chosen and purified by streaking for independent colonies on SD medium supplemented with caspofungin. After incubation of plates, cell mass was deposited at −70 °C. Only one caspofungin-adapted mutant was randomly chosen from each independent experiment to study DNA sequence genome-wide. Our method to obtain mutants due to independent mutational events is presented in [13]. Two of the mutants, SMC60-2-5 and JMC200-3-4, adapted to caspofungin by losing one Ch5 [9]. Two other mutants, JMC160-2-5 and JMC200-2-5, remained euploids [9]. The chromosome conditions of all strains have been extensively characterized [9] and were also confirmed in this study.

Cells were maintained, stored, and grown using our standardized approach that prevents induction of chromosome instability [14]. This approach favors maintaining cells that represent a major fraction in the population of cells (see [15]). Briefly, cells were stored in a 25% (vol/vol) glycerol solution at −70 °C to interrupt metabolism. When needed, cells from a −70 °C stock were streaked for independent colonies on YPD plates and incubated at 37 °C until young colonies of the approximate size of (1–3) × 10^5^ cells/colony grew up. Young colonies were collected, suspended in sterile water, and after proper dilutions, prepared with the aid of a hemacytometer; approximately 3000 colony-forming units were plated and incubated until the appearance of young colonies.

The preparation of yeast-peptone-dextrose (YPD) medium have been described previously [16]. To prepare solid media, 2% (*w*/*v*) agar was added.

### 2.2. DNA-Sequencing and Analysis

Extraction of total DNA was performed as described previously [17]. DNA-seq was performed on three independent batches of cells for each strain, generating 100× average genome coverage. The raw reads were filtered using the program Trimmomatic [18] to remove adapters, as well as low-quality sequences. The filtered reads were mapped to the reference genome (Assembly 22 for the strain SC5314 [19]) using the program NextGenMap [20]. Variant call files were generated through the samtools/bcftools pipeline [21].

The variant calls from each sequencing replicate were combined for each strain and the average fraction of sequencing reads that supported the alternate sequence among the replicates with sequencing data at the variant site was calculated for each variant call (a sample distribution is shown in Figure S1). The variants in each mutant strain were scored based on the difference in the fraction of reads that support the variant in the mutant strain and the parental strain (a sample distribution is shown in Figure S2). A threshold was then applied to those variants to filter those variants with only moderate differences between the mutant and parental strains. This threshold is necessary because *C. albicans* instability results in occurrence of some minor fractions of variants in population of cells [15]. As such, it is possible for a variant to be present in only a portion of the sequenced population of cells. Those variants with a score of ≥0.90 were selected for further analysis. In addition, to identify variants with less rigorous support from the sequencing data, a score threshold of ≥0.66 was used. This score is the maximum score possible when the variant is present in two out of three sequencing runs for an individual strain.

Each variant was then mapped to a genome feature. If the variant was not contained within any annotated feature, the closest feature was determined and associated with the variant if it was within 1kb of the variant site. Gene Ontology (GO) term enrichment analysis on the genes identified was performed using the goatools Python module [22], using GO associations from the Candida Genome Database (CGD) (http://www.candidagenome.org/GOContents.shtml (accessed on 24 June 2019)).

DNA-seq data for SC5314, JRCT1, SMC60-2-5, JMC200-3-4, JMC160-2-5, and JMC200-2-5 are available at SRA with submissionPRJNA433858.

## 3. Results

### 3.1. DNA Changes

In order to search for DNA changes that influence ECN susceptibility, we analyzed DNA-seq data of the four model mutant strains that acquired increased tolerance to caspofungin (JMC160-2-5 and JMC200-2-5 that are normal diploids, and JMC200-3-4 and SMC60-2-5 that are lacking one Ch5). See Table 1 for the origin, relationship, and properties of strains. Both diploid mutants and one aneuploid mutant were analyzed vs. their parental strain JRCT1, while the remaining aneuploid mutant was analyzed vs. its parental strain SC5314. The variant calls for each mutant strain were scored based on the support for the alternate sequence in the reads from the mutant strain versus the parental strain (Section 2). Because the *C. albicans* strains are maintained as a potentially heterozygous population of cells containing a major fraction of cells and some minor fractions [15], we introduced a statistical threshold of 0.90, which requires that a sequence variation be present in at least 90% of the reads (Section 2). The sequence variations that scored ≥0.90 in each mutant strain were analyzed for their position in the genome. In addition to sequence variations that occurred within ORFs, the sequence variation that mapped within 1 kb outside of an ORF was also associated with that ORF. If the nearest ORF was further away than 1 kb, the sequence variation was not associated with any ORF.

**Table 1:** *C. albicans* caspofungin-adapted mutants * and their parental strains.

Strain	Description	Phenotype **	Source
SC5314	Parental strain, normal diploid	Caspofungin susceptible	A.D. Johnson laboratory [23]
SMC60-2-5	Same as SC5314, but Ch5 monosomy, *MTL**α***.	Caspofungin tolerant	[9]
JRCT1	Parental strain, normal diploid	Caspofungin susceptible	Same as above
JMC200-3-4	Same as JRCT1, but Ch5 monosomy, *MTLα*.	Caspofungin tolerant	Same as above
JMC160-2-5	Same as JRCT1, no ploidy change.	Same as above	Same as above
JMC200-2-5	Same as JRCT1, no ploidy change.	Same as above	Same as above

* Note that the mutants acquired decreased caspofungin susceptibility in the absence of *FKS1* mutations [9]. ** See [24] for more details of ECN phenotypes of these caspofungin-adapted mutants.

By applying a statistical threshold of 0.90, we found in total a relatively small number of 112 unique sequence variations (mutations) of which 88 mutations were associated with 43 ORFs (55 mutations mapped within the ORF, 31 mutations within 1 kb of an ORF, and two mapped to introns) (Table S1).

Of the 55 mutations mapping within an ORF, 24 are synonymous substitutions, two frameshifts, one of which resulting in a premature stop codon; and one single codon deletion. The 28 remaining mutations are single nucleotide non-synonymous substitutions. Mutations within 1 kb of ORF occurred more often in the 5′-UTR than in the 3′-UTR by a 1.67:1 ratio (Table S2).

In addition to the 88 sequence variations associated with ORFs, a total of 16 mutations mapped to eight genomic features that were not ORFs: Tca4-4 retrotransposon; Tca2-7 retrotransposon; lambda-4a long terminal repeat; mu-Ra long terminal repeat; MRS-7b Major Repeat Sequence; MRS-R Major Repeat Sequence; RB2-5a repeat sequence; and tL (CAA) leucine tRNA. A further eight mutations were not associated with any ORF or other genomic feature (Tables S1 and S2).

**Figure 1:**
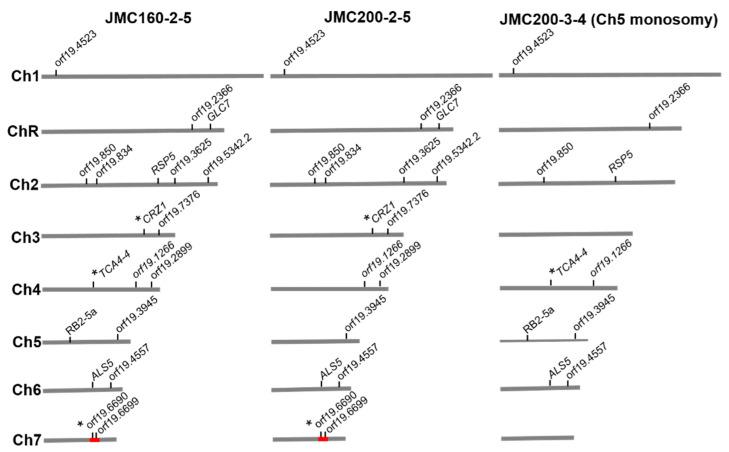
Distribution of shared mutations over chromosomes of caspofungin-adapted mutants JMC160-2-5, JMC200-2-5, and JMC200-3-4. * Indicates the presence of shared mutations in both alleles of a gene or in both copies of a genomic feature. Red block represents a 38 kb mutation prone region on Ch7 (see Figure 2). For more details, see Table 2 and Table S2.

Both diploid mutants, JMC160-2-5 and JMC200-2-5, and one mutant with monosomic Ch5, JMC200-3-4, all originated from the parental strain JRCT1, acquired similar mutational profiles. As summarized in Table 2 and Figure 1, in these profiles, a total of 17 out of 42 identified ORFs with mutations in those mutant strains, as well as one retrotransposon and one repeat sequence, contain mutations in two or in all three adapted mutant strains. Many of such mutations are identical.

The remaining aneuploid mutant SMC60-2-5, which has monosomic Ch5 and originated from a different parental strain, SC5314, is distinct from the above three mutant strains. The mutational profile of SMC60-2-5 contains only one mutation that passed the threshold of 0.90. This mutation is an A→G substitution found 432 bp upstream the start codon of *SOU1* (orf19.2896) on Ch4 (Table S2). The *SOU1* ORF is not mutated in the profiles of the three JRCT1-derived strains.

Next, our analysis focused on mutations that were identified in multiple caspofungin-adapted mutants. Strikingly, the three JRCT1-derived mutants share mutations in a total of six ORFs, residing on various chromosomes that contain identical mutations in all three strains. Specifically, as summarized in Table 2, orf19.1266 (1 nt deletion at position 348), orf19.3945 (1 nt deletion at position 21) and *ALS5* (orf19.5736) (A→C synonymous substitution at position 1488) harbor mutations inside of an ORF. The other three mutations, orf19.2366 (1 nt deletion at position −213), orf19.850 (1 nt deletion 840 nt into the 3′ UTR), and orf19.4523 (4 nt deletion at position −83), are within 1 kb of an ORF. Of special note, five of those six loci are associated with uncharacterized ORFs. There is a further ORF, orf19.6699, which shares a non-synonymous G→A substitution at nucleotide 895 between two of three strains, euploids JMC160-2-5 and JMC200-2-5. Each of the further four ORFs, *GLC7* (orf19.6285), orf19.834, orf19.3625, and orf19.5342.2, has a single mutation outside the ORF (at positions −168, +200, +88, and −140, respectively) that are shared between the euploid strains JMC160-2-5 and JMC200-2-5. A separate mutation was found in orf19.4557 in the euploid strain JMC200-2-5 and aneuploid strain JMC200-3-4 18 nt into the 3′ UTR.

**Figure 2:**
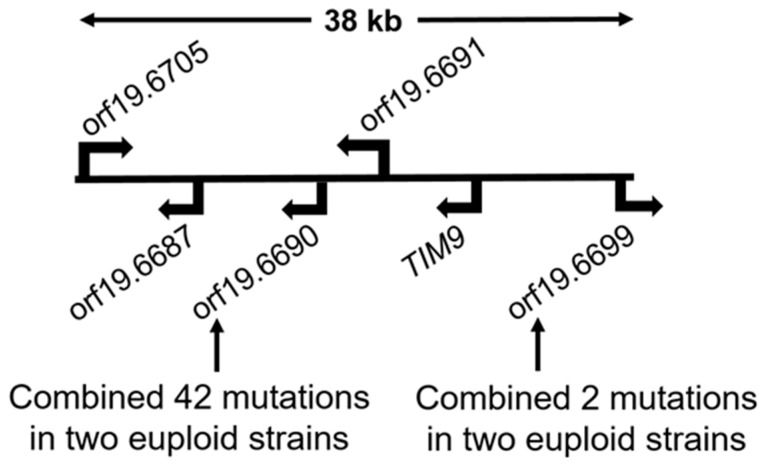
Cartoon of 38 kb region on Ch7 that contains six mutated ORFs. Each orf19.6705, orf19.6687, orf19.6691 and *TIM9* contains two mutations in ORF, three mutations outside of ORF, five mutations outside of ORF, and two mutations in ORF, respectively, in the euploid adapted strain JMC160-2-5. Additionally, orf19.6690 contains 25 mutations in the euploid JMC160-2-5 and 17 mutations in the euploid JMC200-2-5 that are combined in both alleles in each of these two ORFs. The 16 mutations are identical between both euploid strains. Orf19.6699 contains one mutation in each euploid strain JMC160-2-5 and JMC200-2-5.

Another striking result was obtained with orf19.6690 (Table 2 and Figure 2), which harbors 25 mutations across both alleles in one euploid mutant, JMC160-2-5, and 17 mutations combined in both alleles in another euploid mutant, JMC200-2-5, of which 16 mutations, all inside the ORF, are identical between those two mutants. All the mutations are single nucleotide substitutions and occur within the first 102 nt of the 2078 nt ORF. 11 of the 16 mutations in common between the two mutants are non-synonymous (Table 2 and Table S2). Another mutational hot-spot is represented by the 6.9 kb retrotransposon Tca4-4 that contains eight different mutations within a 133 nt site in total between two mutant strains, euploid JMC160-2-5 and aneuploid JMC200-3-4, (Table 2 and Table S2). Another mutational hot-spot is represented by the gene *CRZ1* (orf19.7359) that has four mutations in euploid JMC160-2-5, 3 of which are also present in another euploid JMC200-2-5. Common mutations are represented by synonymous A→G substitutions at nucleotides 96 and 99 and a non-synonymous G→A substitution at nucleotide 121, while an additional mutation in euploid JMC160-2-5 is a non-synonymous substitution A→G at nucleotide 167 (Table 2 and Table S2).

Furthermore, *RSP5* (orf19.3628) has 2 mutations (119 and 120 bases upstream of the start codon) in the euploid strain JMC160-2-5, 1 of which is also found in aneuploid JMC200-3-4. The orf19.7376 has 2 synonymous mutations (an A→G substitution at nucleotide 684 and a T→C substitution at nucleotide 709) inside the ORF in the euploid strain JMC160-2-5, 1 of which (at position 684) is also found in another euploid JMC-200-2-5. The orf19.2899 has 2 mutations (a synonymous A→G substitution at nucleotide 279 and a non-synonymous A→G substitution at nucleotide 314) in the euploid strain JMC160-2-5, one of which (at position 279) is also found in euploid JMC200-2-5. Finally, RB2-5a, which is a repeat sequence of approximately 6 kb, part of Major Repeat Sequence on Ch5, has 2 mutations in aneuploid strain JMC200-3-4, a G→A substitution at nucleotide 1752 and a C→T substitution at nucleotide position 1765. The substitution at nucleotide 1752 is also present in the euploid strain JMC160-2-5.

In the euploid strain JMC160-2-5, there are five additional ORFs with multiple mutations only identified in this strain. There is a single ORF associated with five mutations (orf19.6691), two ORFs associated with three mutations (orf19.6687, orf19.7365), and two ORFs associated with two mutations (orf19.6705, *TIM9* [orf19.6696]) (Table S1, summarized in Table S3). It is of note that four of the five ORFs listed above reside on Ch7 [orf19.6687, orf19.6691, *TIM9* (orf19.6696), orf19.6705], while only the one ORF (orf19.7365) resides on Ch3.

Also of note is that the four ORFs (orf19.6705; orf19.6687; orf19.6691, and *TIM9*) on Ch7 mapped to a single 38 kb region (Figure 2). Furthermore, two ORFs (orf19.6690 and orf19.6699), which are mutated in both euploid strains (JMC160-2-5 and JMC200-2-5), harboring, strikingly, a combined 27 unique mutations, are also found in this Ch7 locus (Figure 2; Table 2). This implies that this 38 kb region is a mutationally prone locus on Ch7.

In the aneuploid strain JMC200-3-4, the ORF *INT1* (orf19.4257) is associated with five mutations (Table S1, summarized in Table S3). The *INT1* gene with multiple mutations in aneuploid JMC200-3-4, resides on Ch5, which carries multiple genes for negative control of ECN susceptibility [7,10].

The rest of the mutations that are not shared among multiple strains are summarized in Table S3.

Single nucleotide non-synonymous substitutions and frameshifts could have a direct effect on protein structure and function. Examples include orf19.2899, in which hydrophobic iso-leucine is substituted by hydrophilic threonine, I105T (Table S4). Additionally, in orf19.6690, there is an array of 17 various substitutions including amino acid with aromatic side chain replaced by amino acid with aliphatic side chain, F10L. In addition, lysine is replaced by asparagine, K16N. Hydrophobic iso-leucine substituted by threonine, I22T. In orf19.6699, glycine is replaced by arginine, G299R. This mutation changes an amino acid with a small side chain and no charge to an amino acid with a large and positively charged side chain. In *CRZ1*, L41F leads to substitution of an amino acid with an aliphatic side chain to an amino acid with an aromatic side chain. In orf19.1606, S169F causes a change of an amino acid with a small polar side chain to an amino acid with a large and hydrophobic side chain. In orf19.6694, I497T changes an amino acid with a hydrophobic side chain to an amino acid with a hydrophilic side chain. Of two frameshifts, one occurs in orf19.1266 close to the C-terminal end of the polypeptide and might not be detrimental for the function. While, another in orf19.3945 leads to a premature termination stop codon that is close to the N-terminal end of polypeptide, thus having a dramatic impact on protein function.

No enrichment of GO terms using CGD (http://www.candidagenome.org/GOContents.shtml (accessed on 24 June 2019)) is found when all 17 ORFs that contained mutations in two or more mutant strains (Table 2) were analyzed with GO, presumably because these are predominantly uncharacterized ORFs.

At a reduced statistical threshold of ≥0.66, there were a total of 214 mutations, 166 of which mapped to 87 ORFs, 29 of which mapped to 16 other genome features, and 16 of which were more than 1000 nucleotides (nts) outside of any ORF or genome feature (Table S2). Notably, at the lower threshold, the distinct aneuploid mutant SMC60-2-5 showed mutations in only two additional ORFs, one repeat sequence, and two more mutations that were not mapped to any genome feature. None of the additional mutated sites in the distinct strain were mutated in the any of the three strains. This analysis confirms a relatively small number of mutations in caspofungin-adapted strains and that mutations in SMC60-2-5 constitute a distinct profile.

Our data demonstrate that *C. albicans* cells with increased tolerance to caspofungin harbor genome-wide DNA changes that occur in a limited number of loci and are predominantly associated with ORFs. Importantly, mutations in some of the loci occurred repeatedly. Most importantly, mutations in some loci or some genome-wide hotspots are in common between different mutant strains.

### 3.2. Comparison of DNA Changes to Previously Reported Expression Changes

We have previously identified differentially expressed genes (DEGs) in four caspofungin-adapted mutants JMC160-2-5, JMC200-2-5, JMC200-3-4, and SMC60-2-5 that are analyzed here for their DNA changes [7].

A list of a total of 43 ORFs that harbored 88 mutations that scored equal or higher than 0.90 (see Section 3.1) was compared with the individual list of DEGs in each of the three caspofungin-adapted JRCT1-derived mutants. We observed that these mutants (JMC160-2-5, JMC200-2-5, and JMC200-3-4) have 34; 16; and 15 mutated genes, correspondingly, of which only eight; two; and three are DEGs (summarized in Table S3). Of the 17 genes that mutated in more than one of these three strains (Table 2), four, two, and two mutated genes are DEGs, correspondingly, in JMC160-2-5, JMC200-2-5, and JMC200-3-4.

At the reduced threshold of mutational significance, with variations that scored at ≥0.66, a total of 87 ORFs that harbored altogether 95 mutations within and 72 mutations outside an ORF (see Section 3.1) were compared with the individual list of DEGs in each of four mutants. We found that the three strains (JMC160-2-5, JMC200-2-5, and JMC200-3-4) with overall greater degree of similarity and originating from the same parent (JRCT1), have a greater number of mutated genes 56, 34 and 37, respectively, among which only 22; 17; and 14 are DEGs.

The distinct aneuploid strain SMC60-2-5 had 8 DEGs (Table S3); however, none of those was the *SOU1* gene with a mutation detected at more stringent threshold (≥0.90) or the three genes with mutations detected at reduced threshold (≥0.66). Both mutational and transcriptomic analyses indicated lack of overlap between SMC60-2-5, and the other three strains, thereby demonstrating its distinct nature, compared to the other three strains.

A substantial number of mutated genes exhibited changes at transcriptional level (Table S3). We also find that most genes sharing mutations between mutant strains acquired transcriptional changes (Table 2 and Table S2). However, there is a lack of a direct correlation between shared or non-shared changes in the DNA sequences of caspofungin-adapted mutants analyzed in here and their DEGs that we published in [7,10]. Some examples include previously uncharacterized gene orf19.5069 that is upregulated in all three strains; however, it harbors an ORF mutation in one adapted strain only (Table S3). Another striking example is *CRZ1* that harbors a mutational hotspot containing non-synonymous substitutions in both euploid mutants, but not in the aneuploid mutant. *CRZ1* is upregulated in one of two euploid mutants, and, unexpectedly, is downregulated in a mutant with a single Ch5 that has its profiles similar to mutants with no ploidy change, a result for which we do not have a simple explanation.

## 4. Discussion

We conducted genome-wide mutational analyses of four caspofungin-adapted mutants of *C. albicans* that arose in independent experiments on plates supplemented with lethal amounts of caspofungin. These mutants are similar in that their caspofungin minimum inhibitory concentration (MIC) increased two–eight fold in the absence of *FKS1* hot-spot resistance mutations and any other mutations in *FKS1*. In this respect, our caspofungin-adapted mutants imitate dozens of clinical isolates that display a wide range of increased MIC values for ECNs without classical *FKS1* mutations for resistance (reviewed in [7]. We consider our caspofungin-adapted mutants as modeling clinical isolates with elevated caspofungin MIC.

Despite four caspofungin-adapted mutants sharing the phenotype of reduced caspofungin susceptibility, the mutants are significantly different in other aspects. Two of four mutants became adapted by a well-established mechanism of loss of one copy of Ch5 [9]. These aneuploid mutants decreased susceptibility to caspofungin but, intriguingly, increased susceptibility to two other ECNs, anidulafungin and micafungin [24], showing that aneuploid mutants are highly specialized and also implying that different ECNs interact with discrete networks of genes in *C. albicans*. Two other adapted mutants that remained euploid, decreased susceptibility to all three ECNs, caspofungin, anidulafungin and micafungin [24]. Furthermore, adaptation of each class of mutants, aneuploids or euploids, is coupled with a distinct pattern of remodeling of the cell wall, an organelle targeted by ECN drugs (*E. Rustchenko*, unpublished data).

The DNA-seq analysis conducted in this work uncovered more differences among the adapted mutants, as each mutant acquired a unique pattern of DNA changes. Three out of four mutant strains contain significantly more sequence mutations, many of which are shared among these mutants, whereas one mutant is distinct, containing, in a sharp contrast, a single mutation in a different gene. The four caspofungin-adapted strains detail two alternative mutational pathways to allow survival on plates supplemented with caspofungin that could possibly reflect different genetic backgrounds.

Taking into account the phenotypic and genotypic differences among the three more similar mutant strains, the similarities in the DNA changes among the mutants are striking. The outstanding observation is that approximately half of the DNA changes occur in hotspots that are distributed over the genome, ranging from two to 25 mutations, and some of the mutations occur in both alleles of certain ORFs (reviewed in Results). Most importantly, approximately half of some mutations and hotspots are shared by two or all three of the more similar adapted mutants. A striking example of this is a 38 kb region on Ch7 with total of 56 mutations that repeatedly occurred in six ORFs residing within the region. Two of these mutated ORFs are shared between two euploid adapted mutants combining altogether 44 mutations. Interestingly, orf19.6690 combining 42 mutations in two euploid mutated strains increased in expression in both euploid strains. Strikingly, another seven mutations are shared between *CRZ1* ORFs in two euploid mutants. *CRZ1* is of interest for this study, as it encodes a calcineurin-regulated transcriptional factor and is involved with drug resistance and cell wall integrity in various fungi [25]. Crz1p plays a partial role in azole and ECN adaptation in *C. albicans*, implicating other downstream effectors of calcineurin (reviewed in [26]). Expression changes of *CRZ1* has been previously demonstrated to be involved in drug response [27]. We find *CRZ1* expression changes in two adapted mutants, one euploid and one aneuploid, apparently overlapping only partially with mutations that occurred in two euploid mutants (see Section 3 for details).

Taken together, repetitive mutations in the same gene, especially when these mutations are shared by independently-generated adapted strains that differ by their Ch5 condition, strongly indicate that these DNA changes do not occur by chance, but rather in response to the drug stress. We interpret changes that are shared by aneuploid and euploid adapted mutants as indication that DNA changes accompany adaptation to the drug. Further studies are needed to better understand how DNA changes relate to the adaptation to ECN drugs and to define the alternative systems governing mutational pathways.

By addressing the question of whether DNA changes affect gene expressions in each of the adapted mutants, we found a poor correspondence between DNA changes and DEGs. However, interestingly, mutated genes being also DEGs prevail among genes that are shared by two or all three of the JRCT1-derived mutant strains. Overall, expression pattern changes do not seem to depend on the mutagenesis pattern, but at least partial overlap could become clearer if individual proteomics of each adapted mutant is performed.

We would like to emphasize the robustness of our results, as we have used highly stringent sets of statistical criteria to define mutations in this study, as well as a stringent approach to define DEGs in our earlier study [7].

## 5. Conclusions

In summary, we initiated the study of genome-wide changes of DNA sequence of the *C. albicans* laboratory mutants that independently adapted to grow in the presence of certain amounts of ECN drugs and that are modeling clinical isolates of *C. albicans* having increased MICs in the absence of *FKS1* mutations. We find limited amount of DNA changes, half of which occur as mutational hotspots that are largely shared by independent adapted mutant strains. The majority of the DNA changes are represented by single-nucleotide substitutions.

We interpret our data as a strong indication that the stressful process of adaptation to cidal caspofungin can be accompanied by genome-wide mutagenesis in a limited number of locations of DNA sequence.

We propose that at least some of the DNA changes can constitute a reservoir of factors influencing ECN susceptibility. However, further studies, including the protein level, are needed to understand the complexity of the relationship between acquired DNA changes and ECN susceptibility.

## Figures and Tables

**Table 2:** Distribution of mutations including common mutations, in 17 indicated genes and in two non-ORF genome features of three model mutants JMC160-2-5, JMC200-2-5, and JMC200-3-4. A distinct mutant strain SMC60-2-5 is not presented here, as it acquired only one mutation in *SOU1* that is not shared by any of the other mutant strains. Note that numbers show the number of mutations in each gene or genome feature in a given mutant. For the two underlined genes and one genome feature, at least one mutant strain contains mutations in both alleles of the gene or both copies of the genome feature. The genes are presented according to the chromosomes, from R to 7, on which they reside.

			Euploid		Ch5 Mono
Gene Name	Gene Identifier	Systematic Name Chromosome	JMC160- 2-5	JMC200- 2-5	JMC200- 3-4
	orf19.2366	CR_06990W R	1	1	1
*GLC7*	orf19.6285	CR_07650W R	1	1	0
	orf19.4523	C1_02020W 1	1	1	1
	orf19.834	C2_03910C 2	1	1	0
	orf19.850	C2_03700W 2	1	1	1
	orf19.3625	C2_08540C 2	1	1	0
*RSP5*	orf19.3628	C2_08500W 2	2	0	1
	orf19.5342.2	C2_10650W 2	1	1	0
*CRZ1*	orf19.7359	C3_05780C 3	4	3	0
	orf19.7376	C3_05950W 3	2	1	0
	Tca4-4	C4_03210C 4	6	0	2
	orf19.1266	C4_05800C 4	1	1	1
	orf19.2899	C4_06360C 4	2	1	0
	orf19.3945	C5_04610W 5	1	1	1
	RB2-5a	C5_01660C 5	1	0	2
	orf19.4557	C6_04120C 6	0	1	1
*ALS5*	orf19.5736	C6_03690W 6	1	1	1
	orf19.6699	C7_03650W 7	1	1	0
	orf19.6690	C7_03580C 7	25	17	0

## Data Availability

DNA-seq data for SC5314, JRCT1, SMC60-2-5, JMC200-3-4, JMC160-2-5, and JMC200-2-5 are available at SRA with submission PRJNA433858.

## Supplementary Materials

The following supporting information can be downloaded at: https://www.mdpi.com/article/10.3390/microorganisms11081870/s1, Figure S1. Distribution of read fractions for variants in JMC160-2-5; Figure S2: Distribution of read fractions differences for variants in JMC160-2-5; Table S1: Mutations associated with ORFs or other genomic features that scored ≥ 0.90; Table S2: Specific mutations associated with ORFs or other genomic features whose scores exceeded the relaxed statistical filter ≥ 0.66 in four C. albicans model mutants JMC160-2-5, JMC200-2-5, JMC200-3-4 and SMC60-2-5; Table S3: Expression changes [7] and distribution of mutations, including common mutations, in 43 indicated genes, eight non-ORF genome features, as well as eight unknown genomic features of four caspofungin-adapted mutant strains JMC160-2-5, JMC200-2-5, JMC200-3-4 and SMC60-2-5; Table S4: Specific mutations associated with ORFs whose scores exceeded statistical filter ≥ 0.90 in four *C. albicans* model mutants JMC160-2-5, JMC200-2-5 and JMC200-3-4.
