# Supplementary material for: Genome-Wide DNA Changes Acquired by Candida albicans Caspofungin-Adapted Mutants
Source: Microorganisms. 2023 Jul 25;11(8):1870. doi: 10.3390/microorganisms11081870 (PMC10458384; doi:10.3390/microorganisms11081870)
Supplement: Supplementary file 1 [file microorganisms-11-01870-s001.zip › Supplemental figure legends.pdf]

Microorganisms

Genome-wide DNA changes acquired by *Candida albicans* caspofungin-adapted mutants.

Jeffrey Zuber, Sudisht K. Sah, David H. Mathews, Elena Rustchenko

Figure legends of Supplemental Fig. S1 and Fig. S2

Figure S1. Distribution of read fractions for variants in JMC160-2-5. For each variant in the mutant strain JMC160-2-5, the fraction of sequencing reads that supported the variant was determined (by dividing the number of reads agreeing with the variant sequence by the total number of reads mapped to the site of the variant). The read fraction was averaged across three independent sequencing runs. Shown here is a histogram of average read fraction distribution across all variants for the strain JMC160-2-5.

Figure S2. Distribution of read fractions differences for variants in JMC160-2-5. For each variant found in either the strain JMC160-2-5 or its parent strain JRCT1, the difference in read fractions (read fraction in the mutant strain subtracted by the read fraction for the same variant in the parental strain) was calculated. Shown here is a histogram of the distribution of the read fraction differences for the strain JMC160-2-5. Those variants that only appear in the mutant strain will appear on the right of the distribution (near 1.0), while those variants that only appear in the parental strain appear on the left of the distribution (near -1.0), and those variants that are in both the parental and mutant strains appear in the center of the distribution (near 0.0).
