# Supplementary material for: Genome-Wide DNA Changes Acquired by Candida albicans Caspofungin-Adapted Mutants
Source: Microorganisms. 2023 Jul 25;11(8):1870. doi: 10.3390/microorganisms11081870 (PMC10458384; doi:10.3390/microorganisms11081870)
Supplement: Supplementary file 1 [file microorganisms-11-01870-s001.zip › Table S3.pdf]

Table S3. Expression changes [7] and distribution of mutations, including common mutations, in 43 indicated genes, 8 non-ORF genome features, as well as 8 unknown genomic features of four caspofungin-adapted mutant strains JMC160-2-5, JMC200-2-5, JMC200-3-4 and SMC60-2-5. Note that numbers show the number of mutations in each gene in a given mutant. For the two genes and one genomic feature in bold, at least one mutant strain contains mutations in both alleles of the gene. The genes are presented according to the number of shared mutations across three more similar mutant strains.

Gene identifier gene name	Chr	Expression ratio mutant/parent strain				No. of mutations in ORF[†] or within 1 kb of ORF‡			
		Euploid		Aneuploid		Euploid		Aneuploid	
		JMC 160-2-5	JMC 200-2-5	JMC 200-3-4	SMC 60-2-5	JMC 160-2-5	JMC 200-2-5	JMC 200-3-4	SMC 60-2-5
orf19.1266	4	NS	NS	NS	**1.91**	1*	1*	1*	0
orf19.3945	5	NS	NS	**0.46**	NS	1*	1*	1*	0
orf19.4523	1	**1.90**	NS	NS	NS	1*	1*	1*	0
orf19.850	2	**0.37**	NS	**0.42**	NS	1*	1*	1*	0
orf19.2366	R	NS	NS	NS	NS	1*	1*	1*	0
orf19.5736 *ALS5*	6	NS	NS	NS	NS	1*	1*	1*	0
orf19.5342.2	2	NS	NS	**0.43**	NS	1*	1*	0	0
orf19.3625	2	NS	NS	NS	NS	1*	1*	0	0
orf19.6285 *GLC7*	R	NS	NS	NS	NS	1*	1*	0	0
orf19.6699	7	NS	NS	NS	NS	1*	1*	0	0
orf19.834	2	NS	NS	NS	NS	1*	1*	0	0
**orf19.6690**	7	**2.01**	**2.09**	NS	**1.68**	25[#]	17[#]	0	0
**orf19.7359 *CRZ1***	3	NS	**1.64**	**0.46**	NS	3*+1	3*	0	0
orf19.2899	4	NS	NS	NS	NS	2	1	0	0
orf19.7376	3	NS	NS	NS	NS	2	1	0	0
orf19.3628 *RSP5*	2	NS	NS	NS	NS	2	0	1	0
orf19.801 *TBF1*	2	**0.18**	**0.49**	NS	NS	1	0	0	0
orf19.4557	6	NS	NS	NS	NS	0	1	1	0
orf19.2896 *SOU1*	4	NS	NS	NS	NS	0	0	0	1
orf19.4257 *INT1*	5	**0.17**	NS	NS	NS	0	0	5	0
orf19.6691	7	**0.30**	**0.37**	NS	NS	5	0	0	0
orf19.6687	7	NS	NS	NS	NS	3	0	0	0
orf19.7365	3	NS	NS	NS	NS	3	0	0	0
orf19.6696 *TIM9*	7	NS	**0.63**	NS	**0.57**	2	0	0	0
orf19.6705	7	NS	NS	NS	NS	2	0	0	0
orf19.1497 *ZCF6*	2	NS	NS	NS	**0.42**	0	0	1	0
orf19.4330	5	NS	NS	**0.50**	NS	0	0	1	0

orf19.1606	3	NS	**1.93**	**0.44**	NS	1	0	0	0
orf19.1980 *GIT4*	5	NS	NS	NS	**0.25**	1	0	0	0
orf19.2014 *BCY1*	2	**2.10**	**1.65**	NS	NS	1	0	0	0
orf19.4476	1	**1.86**	NS	NS	**3.20**	0	0	1	0
orf19.4991 *MPT5*	1	**3.71**	NS	NS	NS	0	0	1	0
orf19.5069	1	**3.33**	**2.60**	**2.11**	NS	1	0	0	0
orf19.6689 *ARG4*	7	**0.39**	**0.48**	NS	**2.93**	1	0	0	0
orf19.6993 *GAP2*	3	NS	NS	**0.32**	NS	1	0	0	0
orf19.6994 *BAT22*	3	NS	NS	**1.55**	**1.98**	1	0	0	0
orf19.1177	6	NS	NS	NS	NS	0	0	1	0
orf19.6986	3	NS	NS	NS	NS	0	0	1	0
orf19.4215 *FET34*<br>orf19.6693<br>orf19.6694,<br>orf19.6985 *TEA1*<br>orf19.2850	6<br>7<br>7<br>3<br>R	NS	NS	NS	NS	1	0	0	0
Genomic Feature									
RB2-5a	5	NA	NA	NA	NA	1*	0	1+1*	0
**TCA4-4**	4	NA	NA	NA	NA	6	0	2	0
Tca2-7,<br>MRS-R,<br>tL(CAA)2	7<br>R<br>2	NA	NA	NA	NA	0	0	1	0
mu-Ra,<br>lambda-4a,<br>MRS-7b	R<br>4<br>7	NA	NA	NA	NA	1	0	0	0
Unknown Genomic Feature with Chr coordinates									
1A-1656266	1	NA	NA	NA	NA	0	0	0	1
4B-1526536	4	NA	NA	NA	NA	0	0	1	0
2B-2040120	2	NA	NA	NA	NA	1	0	0	0
RA-1884835	R	NA	NA	NA	NA	0	0	1	0
RB-1288392	R	NA	NA	NA	NA	0	0	1	0
4A-1299664,<br>4B-1299656,<br>RA-1884702	4<br>4<br>R	NA	NA	NA	NA	1	0	0	0

† Black color stands for mutation harbored within ORF

‡ Green color stands for mutation harbored within 1 kb of ORF

* Stands for identical mutations

# Stands for 16 identical mutations that are shared between adapted strains.

NS stands for No Significant change

NA stands for Not Applicable
