# Supplementary figures and images for: Genome-Wide DNA Changes Acquired by Candida albicans Caspofungin-Adapted Mutants
Source: Microorganisms. 2023 Jul 25;11(8):1870. doi: 10.3390/microorganisms11081870 (PMC10458384; doi:10.3390/microorganisms11081870)

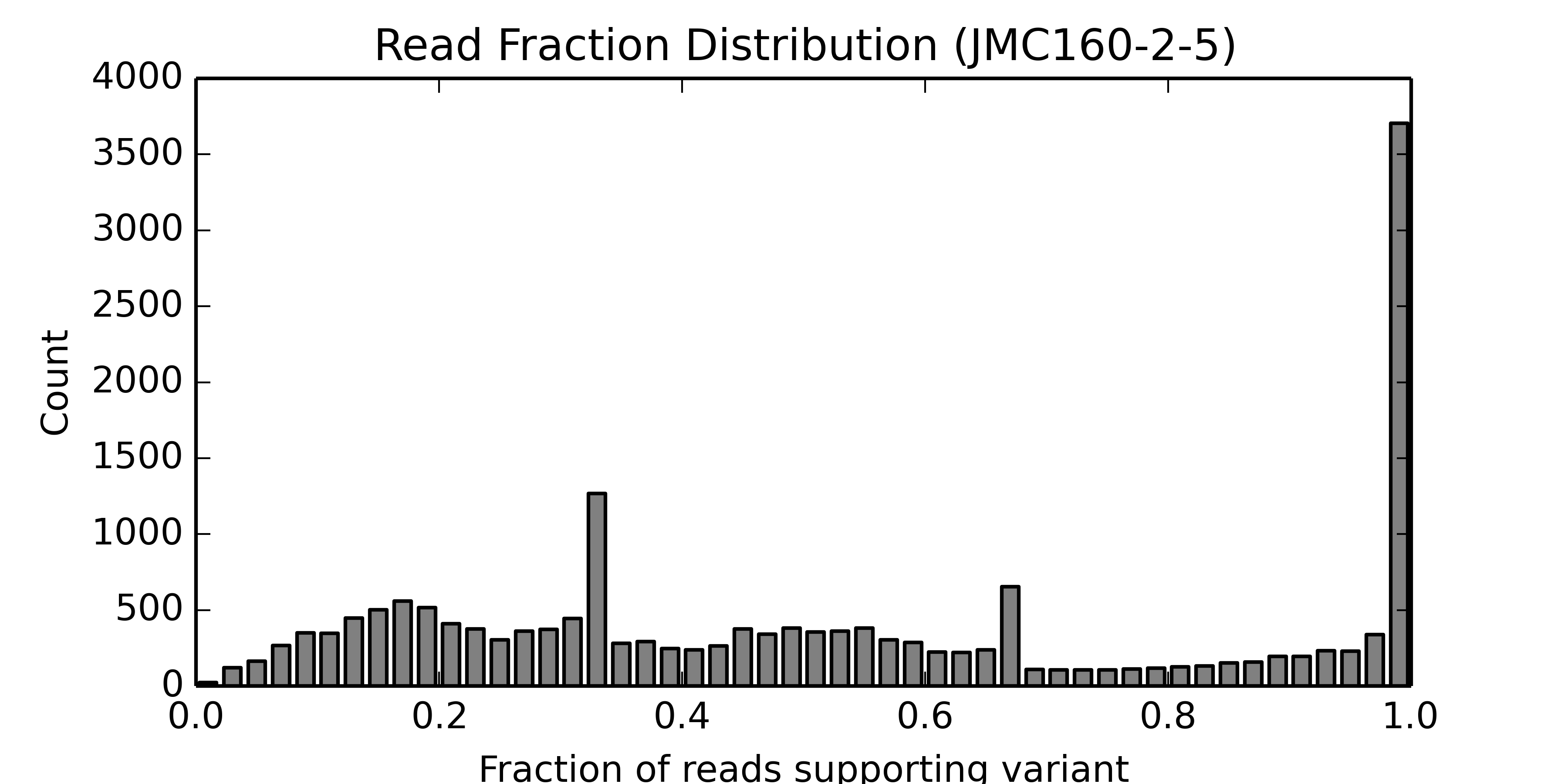

Supplement: Supplementary file 1 [file microorganisms-11-01870-s001.zip › Fig S1.png]

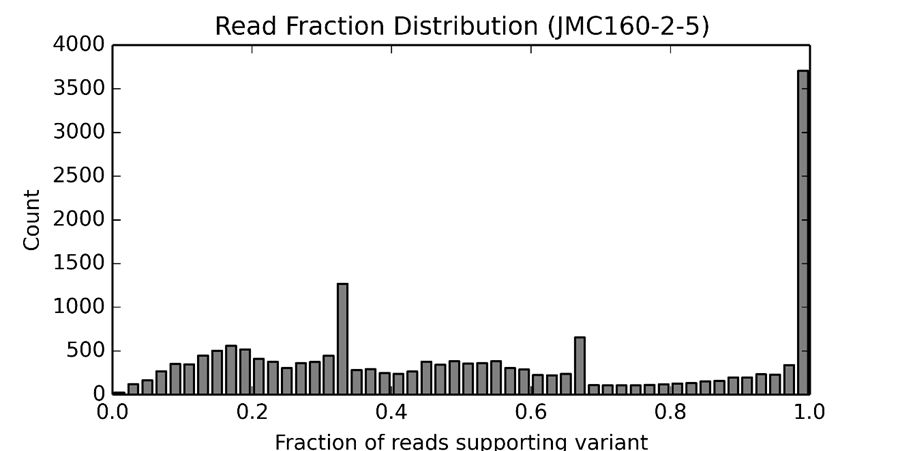

Supplement: Supplementary file 1 [file microorganisms-11-01870-s001.zip › FIG S1.tif]

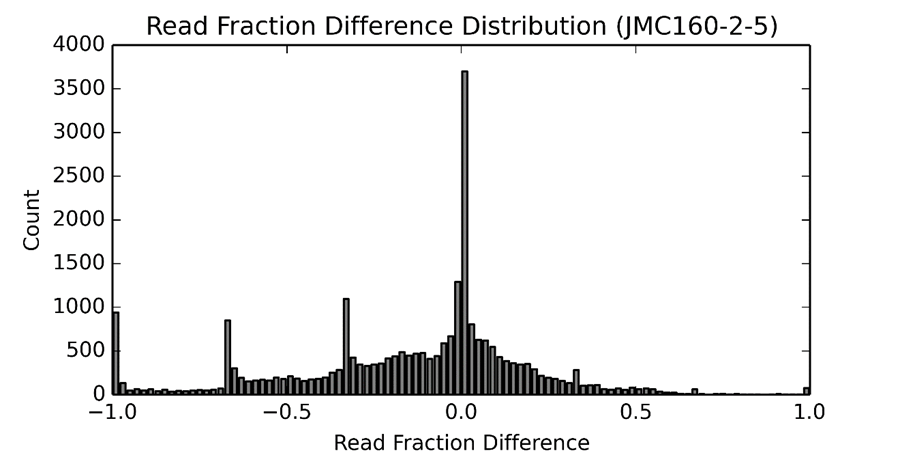

Supplement: Supplementary file 1 [file microorganisms-11-01870-s001.zip › FIG S2.tif]
